# Controlling Myopia: Insights from a Nation-Wide Clinical Practice Survey

**DOI:** 10.3390/jcm15114237

**Published:** 2026-05-30

**Authors:** Aldo Vagge, Gabriele Drago, Carlo Catti, Serge Resnikoff, Roberto Caputo, Paolo Nucci, Lelio Sabetti, Matteo Gabriele Gabutto, Massimiliano Serafino, Giuliano Stramare, Irene Schiavetti, Maurizio Fedriga, Maria Musolino, Michele Iester

**Affiliations:** 1 Istituto di Ricovero e Cura a Carattere Scientifico Ospedale Policlinico San Martino, 16132 Genoa, Italy; 2 Clinica Oculistica, Dipartimento di Neuroscienze, Riabilitazione, Oftalmologia, Genetica e Scienze Materno-Infantili, Università di Genova, 16132 Genoa, Italy; 3 Dipartimento di Medicina Sperimentale, Università di Genova, 16126 Genoa, Italy; 4 School of Optometry and Vision Science, University of New South Wales, Sydney, NSW 2033, Australia; 5 Brien Holden Vision Institute, Sydney, NSW 2052, Australia; 6 Pediatric Ophthalmology Unit, Meyer Children’s Hospital, 50139 Florence, Italy; 7 Department of Biomedical, Surgical and Dental Sciences, University of Milan, 20122 Milan, Italy; 8 Department of Biotechnological and Applied Clinical Sciences, University of L’Aquila, 67100 L’Aquila, Italy; 9 Istituto di Ricovero e Cura a Carattere Scientifico Istituto G. Gaslini, 16147 Genoa, Italy; 10 Istituto di Ricovero e Cura a Carattere Scientifico Sacrocuore Don Calabria Hospital, 37024 Negrar di Valpolicella, Italy

**Keywords:** myopia, survey, atropine, peripheral defocus lenses

## Abstract

**Background/Objectives:** To evaluate the knowledge and strategies employed by Italian ophthalmologists and orthoptists in control myopia progression. **Methods:** A comprehensive survey was developed, consisting of 26 questions focusing on various aspects of myopia progression control, and it was distributed electronically using the Google Forms platform to all members of the major ophthalmological and orthoptist societies (S.I.S.O. ETS, AIOrAO, AIMO, and SIOPS). **Results:** A total of 662 respondents were obtained. 68.88% of participants use myopia progression control into their clinical practice, and 78.1% feel adequately updated in this field. Myopia-defocus lenses emerge as the first-line method for controlling myopia progression in approximately 43.05% of cases, whereas 33.53% recommend only behavioral methods. Atropine is used at a concentration of 0.01% as the first choice (55.59% of responses). Among myopia-defocus technologies, 38.82% use DIMS lenses, while another 38.82% leave the choice to the optician. Peripheral defocus lenses and low-dose atropine are considered scientifically valid by a larger number of participants (79.61% and 58.31%, respectively). **Conclusions:** This survey indicates that many ophthalmologists consider themselves well-versed and updated in current management approaches. Peripheral defocus lenses and low-concentration atropine emerge as the most widely implemented therapeutic strategies, supported by the perceived strong scientific evidence for their efficacy.

## 1. Introduction

Myopia represents the most prevalent refractive error worldwide, with an alarming increase in incidence, particularly among pediatric and adolescent populations in the last few decades [1,2,3,4]. This epidemiological shift has drawn increasing attention within the ophthalmic community, particularly due to its long-term implications for ocular health and the healthcare systems of various nations. In Europe, and especially in Italy, the rising trend of myopia-affected patients has been recognized as a rising public health concern, with predictions of a continuously increasing rate in prevalence [5,6].

Myopia is primarily associated with axial elongation of the eye, a structural change that greatly increases the risk of different sight-threatening ocular diseases influenced by myopia itself. Those complications include retinal detachment, myopic maculopathy, retinoschisis, choroidal neovascularization, and an increased predisposition to open-angle glaucoma [7,8,9,10]. Given these associations, the early identification and effective management of myopia are pivotal in reducing future ocular diseases uprising. Furthermore, the progression of axial length is often silent and irreversible, making proactive monitoring essential, especially in pediatric populations. The cumulative risk of myopia-related complications, such as retinal detachment, maculopathies and retinoschisis, increases substantially with each diopter of myopic shift, reinforcing the clinical importance of timely intervention. Therefore, integrating routine axial length measurement into clinical protocols may enhance the early detection of progressive myopia. Myopia screening and evaluation are gaining relevance in the field of rare diseases as well, particularly due to the high correlation between myopia and rare conditions such as collagenopathies. Reduced visual acuity is often the first reason of referral to clinicians and therefore the gateway to further investigation of possible syndromic conditions. Preventive strategies should also be emphasized, particularly in high-risk children with familial predispositions, to prevent long-term visual morbidity.

In response to the growing clinical need, the European Society of Ophthalmology (SOE), in partnership with the International Myopia Institute (IMI), has released comprehensive, consensus-driven clinical guidelines. These guidelines emphasize evidence-based strategies for the monitoring and control of myopia progression, standardizing approaches across different healthcare settings [11,12].

Currently, the myopia management strategies encompass a wide range of interventions, including optical, pharmacological, and behavioral treatments [13,14,15,16]. Optical interventions have developed into orthokeratology, multifocal soft contact lenses, and newly developed spectacle lenses designed to modulate peripheral retinal defocus, due to its relevance in the retinal and choroidal remodeling of the eyeball during growth [17,18]. Of note, among pharmacological options it is known that low-concentration atropine eye drops—typically in concentrations between 0.01% and 0.05%—have demonstrated statistically significant efficacy in reducing axial elongation rates and refractive error progression in children and adolescents [19,20,21,22,23,24].

Lifestyle-based interventions are gaining attention and are increasingly recognized for their role in myopia control. These include recommendations such as encouraging children to spend a minimum of two hours per day outdoors and maintaining an appropriate working distance (exceeding 30 cm) during close-up tasks such as reading or screen use [25,26,27,28,29].

Given the expanding body of research and clinical protocols, the integration of these interventions must be tailored to individual patient profiles, considering variables such as age, rate of myopia progression, familial predisposition, and behavioral habits [30].

The surge in screen time due to digital learning environments, especially post-pandemic, has further exacerbated myopia progression among infants [1,2,3,4]. Indeed, the digital-based learning shift emphasizes the importance of improving public health campaigns aimed at promoting good visual habits. Of note, clinicians are also encouraged to engage families in educational activities to encourage preventive routines. Integrating regular visual assessments in school-based health programs and screening events could increase the earlier identification of at-risk individuals. The implementation of these strategies, in combination with clinical interventions whenever needed, may enhance early detection and treatment efficacy, potentially reducing the overall future burden of myopia in the general population.

Despite these progressions, there is a notable scarcity of data regarding awareness, clinical practice, and the therapeutic approaches employed by both ophthalmologists and orthoptists in Italy. The present survey-based study was designed to systematically assess the current knowledge, clinical practices, and opinions of Italian ophthalmologists and orthoptists regarding myopia management and progression control, aiming to respond to this unmet need. We aim to identify gaps in practice and potential areas for educational and policy intervention to optimize the standard of care in myopia control at the Italian national level.

## 2. Materials and Methods

This cross-sectional study was conducted through a collaborative effort between the major Italian ophthalmological and orthoptist societies (Società Italiana di Scienze Oftalmologiche (S.I.S.O. ETS, Rome; Italy), Associazione Italiana Ortottisti ed Assistenti in Oftalmologia (AIOrAO; Messina, Italy), Associazione Italiana Medici Oculisti (A.I.M.O.; Rome, Italy) and Società Italiana di Oftalmologia pediatrica e Strabismo (S.I.O.P.S.; Milan; Italy) and was coordinated by the University Eye Clinic of Genoa, DINOGMI, IRCCS San Martino Polyclinic Hospital. The present study was conducted in accordance with the tenets of the Declaration of Helsinki and followed good clinical practice guidelines.

A comprehensive survey consisting of 26 questions was developed to cover the different aspects of myopia progression control. The questionnaire was distributed electronically to all members of participating societies (S.I.S.O. ETS, AIOrAO, A.I.M.O. and S.I.O.P.S.) using the Google Forms platform (Google LLC, 1600 Amphitheatre Parkway, Mountain View, CA, USA), which allowed for anonymous participation and efficient data collection. The survey platform was chosen to ensure ease of access and completion for participants while maintaining their anonymity.

The questionnaire was structured to collect information across several domains:

- Demographic and professional characteristics (including years of experience, work context, and education level);
- General perception and approach;
- Monitoring and assessment practices;
- Treatment strategies;
- Implementation barriers.

The survey was developed following a multi-step process. First, a panel of senior experts reviewed the items for face and content validity. Subsequently, a pilot test was conducted with eight professionals to ensure the questions were unambiguous and the digital platform functioned correctly. No major structural changes were required after the pilot phase. Due to the anonymous, cross-sectional design, test–retest reliability was not assessed, which is consistent with similar exploratory surveys in the field.

**Patient and Public Involvement**

Patients and the public were not directly involved in the design, recruitment, or conduct of this study. However, the study was designed to reflect the real-world clinical experience and practice of eye-care professionals across Italy, with the aim of ultimately improving patient care in the field of myopia management. The findings of the survey may inform future strategies that more directly engage patients and their families in the development and implementation of myopia control programs.

**Statistical Analysis**

A chi-square test was performed to examine relationships between variables. Statistical significance was set at *p* < 0.05. The analysis focused on identifying patterns and differences in responses linked to years of professional experience, work context (private practice, academic, or hospital settings), education level, and the frequency of myopia discussions with patients. Data was organized into five main analytical tables examining the relationships between these professional characteristics and various aspects of myopia management practices. Results are presented as frequencies and percentages, with statistical significance noted for observed differences between groups. To account for multiple comparisons, a Bonferroni correction was considered; however, all primary findings remained significant at the adjusted level.

## 3. Results

**Demographics and Professional Characteristics:**

The questionnaire, conducted between May to November 2024, was emailed to approximately 5500 members. A total of 662 (12%) members from across different professional categories, including orthoptists (n = 52; 7.85%), specialized/staff practitioners (n = 593; 89.58%), and residents (n = 14; 2.15%), completed the survey. Participants were stratified by years of experience: 0–5 years (n = 50; 7.6%), 6–10 years (n = 61; 9.2%), 11–20 years (n = 124; 18.7%), and >20 years (n = 427; 64.5%). Practice settings included private (n = 407; 61.5%), academic (n = 40; 6.0%), and hospital-based (n = 215; 32.5%) contexts. A minority of professionals (n = 53; 8.01%) did not declare a definite practice setting probably because of the possibility in Italy to practice in different settings at the same time (Table 1).

**General Perception and Approach:**

**Figure 1:**
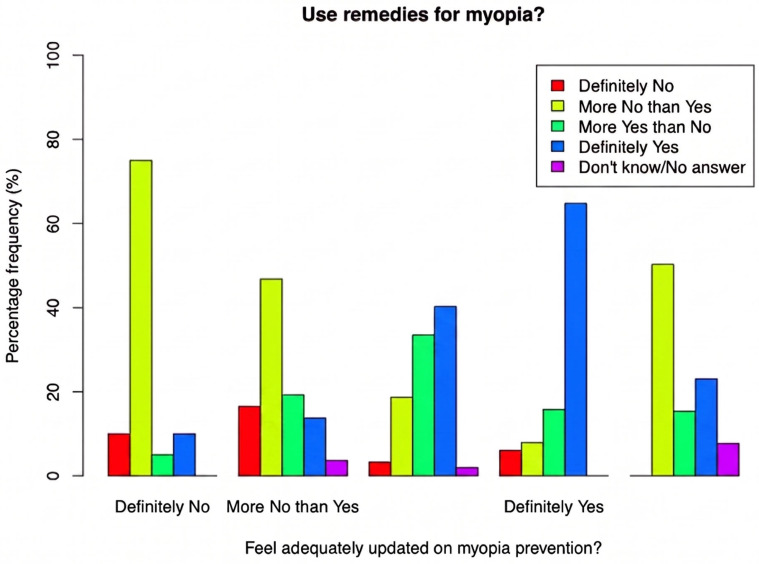
Analysis based on the “Feel adequately updated on myopia prevention” subcategories showing that those who answered “definitely yes” use remedies for controlling myopia progression.

A significant majority of practitioners (*p* < 0.001) demonstrated strong engagement with myopia control methods, with 70.23% “Definitely” implementing myopia control strategies (Figure 1). This commitment varied notably across different practice frequencies, with those discussing myopia in more than 75% of their consultations showing the highest engagement (80.36% “Definitely” using remedies).

The perception of myopia as an increasing concern showed a clear gradient across professional experience levels. Among practitioners with more than 20 years of experience, 49.41% “Definitely” perceived myopia as an increasing problem, while this rose to 60% among those with 0–5 years of experience (*p* = 0.074). This generational difference in perception was particularly evident in academic settings, where 45% of practitioners strongly acknowledged the increasing prevalence of myopia.

**Figure 2:**
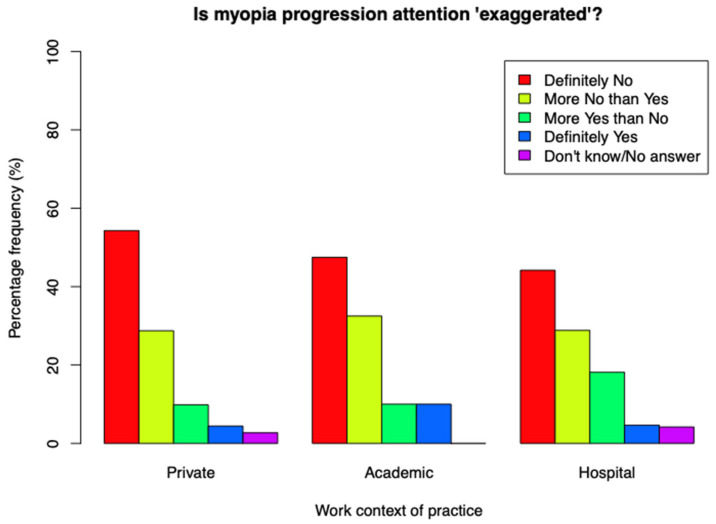
Analysis based on work context regarding practice showing that those working in private, academic and hospital contexts do not think that attention to myopia progression is exaggerated.

The survey also explored practitioners’ views on whether attention to myopia progression was overestimated: significantly (*p* = 0.046), the largest percentage of those working in academic (47.5%), as well as those working in private (54.3%) or hospital (44.19%) settings, agree that attention to myopia control methods is not exaggerated (Figure 2).

Practice patterns showed substantial variation based on the frequency of myopia discussions in clinical settings. Practitioners who discussed myopia in more than 75% of their consultations in daily clinical practice were significantly more likely to express stronger confidence in management strategies (*p* < 0.001), implement comprehensive treatment approaches (80.36% vs. 28.38% in low-frequency groups), and utilize multiple intervention strategies simultaneously (82.14% favoring combined treatments).

Practitioners who “Definitely” perceived myopia as an increasing concern were more likely to adopt evidence-based management approaches (*p* < 0.001), integrate multiple treatment modalities (75.35% vs. 58.72% in less concerned groups), and engage in regular monitoring and assessment (67.44% opting for 6-monthly reviews) (Table 2). Academic centers showed the highest rates of preventive measures (72.5% suggesting lifestyle changes for early intervention), while private practices demonstrated more varied approaches.

**Monitoring and Assessment Practices:**

**Figure 3:**
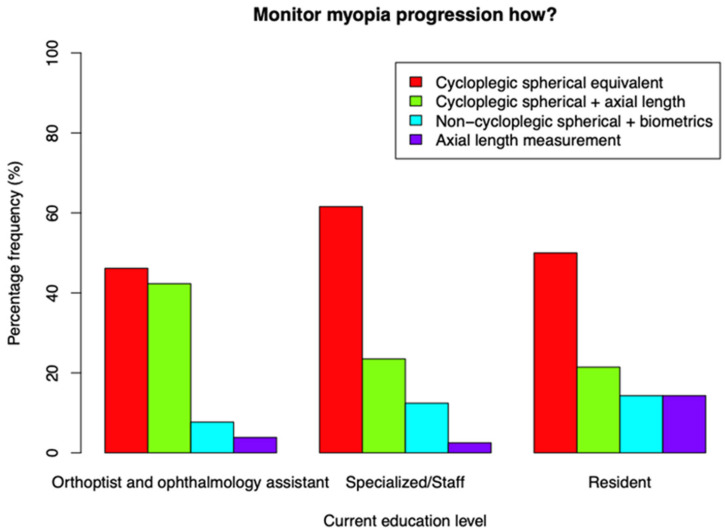
Analysis based on the answers to the question “How is myopia progression monitored?” depending on “Current level of education”, showing that both orthoptists, specialized staff and residents use a cycloplegic spherical equivalent to monitor myopia progression.

Cycloplegic refraction frequency showed significant variation (*p* < 0.001), with most practitioners (55.03%) performing one every 12 months. Overall, the preferred method for monitoring myopia progression was cycloplegic SE (spherical equivalent), as reported by 61.58% of specialized/staff practitioners (*p* = 0.01) (Figure 3).

The choice of monitoring methods showed significant variation according to practice setting (*p* = 0.01) and practitioner experience (*p* = 0.003). The data revealed several distinct patterns:

1. Cycloplegic Spherical Equivalent Monitoring:
  ○ Predominant choice among specialized/staff practitioners (61.58%);
  ○ Higher adoption in private practice settings (60.2%);
  ○ Preferred by early-career practitioners (74% in 0–5 years group).
2. Combined Assessment Approaches (cycloplegic spherical and axial length measured by ocular biometry):
  ○ Cycloplegic spherical with axial length measurements was favored by 32.48% of those facing myopia control at a high frequency, determined as 50–70% of their clinical practice;
  ○ Academic centers showed the highest rate of combined assessment methods (32.5%);
  ○ Mid-career practitioners (11–20 years) demonstrated a lower propensity to use combined approaches (25.81%) compared to using the SE alone.

**Treatment Strategies:**

The survey revealed significant variations in preferred myopia control methods (*p* < 0.001). As a primary intervention, myopic defocus glasses emerged as the leading intervention (43.62% among specialized staff) (Table 2), while behavioral advice was the second most common approach (33.72%), and low-concentration atropine was prescribed by 10.57% of practitioners. When initial strategies proved insufficient, 72.79% of practitioners opted to combine treatments rather than switch approaches. Treatment combinations showed significant variation by practice setting (*p* = 0.039).

**Implementation barriers:**

The study revealed complex and interrelated barriers to implementing various myopia management strategies, with significant variations across different practice settings, experience levels, and treatment modalities.

For low-dose atropine eye drop usage, insufficient knowledge (*p* = 0.037 across practice settings), compounding preparation concerns (*p* = 0.019) and lack of guidelines (*p* = 0.001) were reported as barriers. Regarding myopic defocus lenses, knowledge gaps were more prevalent among less experienced practitioners (*p* = 0.002) whereas cost considerations varied significantly by years of experience (*p* = 0.006), with early-career practitioners reporting higher cost concerns.

Bacterial keratitis risk was a major concern (67.52% and 46.43% among those addressing myopia control, with response rates of more than 50% and 75% respectively in different clinical practices) for orthokeratology (*p* = 0.028).

## 4. Discussion

The growing interest in the control of myopia development and progression represents a pivotal topic in contemporary ophthalmic research and clinical practice. Its importance has significantly increased over the past decade, driven by epidemiological predictions and the possible long-term consequences of out-of-control myopic progression for both visual health and healthcare resources. To date, it remains unclear how frequently ophthalmologists are confronted with this issue in their routine clinical activities and to what extent they are updated regarding the latest evidence-based strategies and therapeutic options in the current clinical setting. Although a considerable increase in research output has been observed in recent years, the scientific literature still lacks robust, large-scale, and long-term comparative studies investigating this to provide certain and standardized guidance on the most effective treatment options for slowing down myopic progression. Furthermore, the variability in clinical practice and access to newer technologies, the economic burden, and differences in healthcare policies across different regions further complicates the translation of research findings into universally accepted clinical protocols and consistent decision-making frameworks. This study aims to offer a detailed overview of awareness, perceptions, and behaviors surrounding myopia management within the Italian ophthalmological community. The high percentage of clinicians reporting active involvement in myopia control (70.23%) underscores a strong recognition of the condition as a pressing public health issue. Notably, this awareness appears more prevalent among early-career professionals, mirroring similar findings from international studies and confirming that the topic has gained significant attention in recent educational protocols and postgraduate training programs [30,31,32,33].

The distribution of respondents, with a significant prevalence of ophthalmologists (90%) over orthoptists (10%), is reflective of the Italian regulatory environment. In Italy, the orthoptist’s scope of practice is centered on functional evaluation and instrumental diagnostics; however, orthoptists are not legally authorized to prescribe pharmacological therapies or optical corrections. Given that the survey was heavily focused on prescriptive management strategies, it likely held less appeal for orthoptists, resulting in a lower participation rate. Nonetheless, their contribution remains vital in the multidisciplinary management of myopia, particularly regarding biometry and the monitoring of binocular function.

Our data align with the global trend of embracing new evidence-supported treatment options. Of note, our findings are consistent with those of Wolffsohn et al. [30], supporting the increasing acceptance of peripheral defocus spectacles as a viable first-line therapy. Interestingly, our cohort of Italian practitioners appeared to be keener to implement these newer lens technologies without initial reliance on traditional corrective approaches, reflecting a growing confidence in the efficacy of novel optical interventions. Therefore, this result may reflect both the diffusion of recent scientific evidence and practical clinical experiences, suggesting good patient tolerance and thus compliance and ease of implementation. In addition, the generational divide in terms of perception and clinical approach is particularly telling. The disproportionate representation of senior practitioners (over 20 years of experience) reflects the current demographic of the professional boards surveyed. While this imbalance precludes a robust inferential comparison between generations, the data provide a realistic descriptive overview of the prevailing clinical trends. Furthermore, while geographic data were not the focus of this study, it should be noted that the Italian market for myopia control (both pharmacological and optical) is highly standardized, with uniform resource accessibility across the Northern and Southern regions. Finally, the lack of practice volume data represents a limitation that future studies should address to better correlate clinical choices with patient flow.

Among professionals with fewer than five years of experience, 60% demonstrated high disease awareness and integration of myopia control strategies, compared to 49.41% in the group with over 20 years of practice. Of note, this may reflect both an evolving educational framework and the role of digital learning tools, which have become more prominent since the COVID-19 pandemic in 2020. Moreover, the widespread adoption of webinars, online CME programs, telemedicine and virtual conferences seems to have significantly contributed to increased exposure to up-to-date guidelines among younger practitioners, who are also digital natives and therefore more compliant and familiar with these digital formats. Additionally, many younger professionals may have experienced, either first-hand or within their peer group, the lifestyle-related risk factors for myopia development, such as daily close-up work and digital device screen use, along with reduced outdoor activity—potentially enhancing their awareness of this clinical issue [34]. In addition, the novel widespread range of treatment options allows for a more patient-tailored management strategy based on their needs and clinical presentation. Consequently, there is a growing demand for standardized protocols to ensure uniformity in clinical practice and outcome assessment. Importantly, continued professional development and interdisciplinary collaboration are essential to maintain high-quality care in this evolving field. This underscores the relevance of integrating updated research findings into routine ophthalmic education and training. Effectively, the combination of optical, behavioral, and pharmacological approaches offers a multimodal framework that is adaptable to different clinical settings. Notably, younger clinicians may be keener to adopt innovative technologies, which could accelerate the clinical translation of emerging research.

A noteworthy observation is the higher degree of awareness and engagement reported within academic institutions, where 45% of respondents showed a more advanced understanding of currently available management options. This result underscores the importance of continuous academic input in keeping clinical attitudes and behaviors updated according to the most recent guidelines. Our survey also highlights a potential gap in ongoing education for clinicians in non-academic settings, suggesting the need to promote educational events, such as inter-institutional workshops, expert-led webinars, and collaborative initiatives to fill this gap. Improving partnerships between academic centers and peripheral healthcare providers could facilitate knowledge transfer and enhance consistency in clinical daily practice. Structured mentorship programs, the integration of evidence-based updates into professional training, and promoting participation in continuing medical education may also enhance engagement. Reducing this educational gap, as highlighted through our survey, would help align practice standards, ensure a more uniform adoption of myopia prevention and control strategies, and contribute to better long-term outcomes for patients across all care settings.

The analysis of treatment preferences revealed that peripheral myopic defocus lenses are the most commonly adopted first-line strategy (43.05%), followed by lifestyle modifications (33.53%) and low-concentration atropine therapy (10.73%). This prioritization likely reflects both the efficacy profile of these interventions and considerations regarding patient compliance. While lifestyle interventions are critical for primary prevention, clinicians are often skeptical about patients’ ability to make and maintain significant behavioral changes, particularly in pediatric populations. Consequently, optical approaches that simultaneously correct refractive error and modulate peripheral defocus are viewed as practical and low-barrier solutions. The data showing that a significant proportion of practitioners (38.82%) leave the specific choice of myopia-defocus lenses to the optician highlights a collaborative practice model prevalent in Italy. Rather than indicating a lack of specific product knowledge, this reflects a professional synergy where the ophthalmologist provides the clinical indication for myopia control, while the optician—leveraging technical expertise in frame fitting and lens geometry—is entrusted with selecting the most appropriate commercial solution for the individual patient. Atropine, despite its proven efficacy, is often deprioritized due to concerns about side effects, limited access to low concentrations, and off-label regulatory considerations in certain regions. At the time of data collection, low-dose atropine was exclusively available in Italy through compounding pharmacies. Practitioners typically prescribed concentrations of 0.01%, 0.02%, or 0.05% based on individual patient needs and the existing literature. While standardized commercial preparations are currently entering the Italian market, the use of galenic formulations remains a consolidated practice.

Orthokeratology, while effective, remains underutilized, likely due to higher costs, increased complexity, and lower adherence rates. Respondents who frequently manage progressive myopia report a preference for closer monitoring (every 6 months), often using cycloplegic refraction and biometric assessments. This group also tends to prioritize defocus lenses as the initial treatment, supplemented by environmental interventions and pharmacologic agents when appropriate.

Subgroup analysis confirmed that practitioners with greater self-reported knowledge were more likely to adopt advanced, evidence-based approaches, such as defocus lens technologies, while clinicians across all practice settings and levels of experience demonstrated comparable attitudes regarding follow-up frequency and general treatment protocols.

Practice patterns showed a strong correlation between familiarity with current evidence and the sophistication of the clinical approach. Eye care professionals who regularly counsel patients on myopia progression displayed greater confidence and were more inclined to implement multimodal strategies, which the literature increasingly associates with improved clinical outcomes [14].

Monitoring approaches varied slightly across settings. While cycloplegic refraction remains the mainstay (61.58%), a trend toward the integration of ocular biometry—particularly axial length measurements—was observed, especially within academic centers (32.5%). This evolution reflects growing awareness of the value of objective biometric data in evaluating disease progression and treatment efficacy. As biometric technologies become more accessible and cost-effective, their incorporation into standard protocols is anticipated to become more widespread [35].

This study is subject to limitations. First, the present manuscript may include potential sampling bias, particularly the underrepresentation of difficult-to-reach practitioners living in rural countryside areas or practicing in smaller private settings. In addition, there is a remote possibility of the duplication of survey responses from members affiliated with multiple societies. Finally, these elements may be a limitation to the generalizability of our results to the entire Italian ophthalmologic workforce.

## 5. Conclusions

This survey offers valuable insights into current practices and perceptions of myopia management in Italy. Encouraging trends in the adoption of evidence-based strategies were observed, especially among younger and academically affiliated professionals. The results support the need for continued professional education, the dissemination of standardized guidelines, and broader access to emerging technologies. These steps are essential to reduce the clinical and economic burden of progressive myopia and ensure consistent, high-quality care across diverse clinical environments.

## Figures and Tables

**Table 1:** Demographics and Practice Characteristics.

Questions	Frequency
Professional categories	
Ophtalmology assistants	52 (7.85%)
Residents	14 (2.11%)
Specialized/Staff pratictioners	539 (89.58%)
NA	3 (0.45%)
Years of experience	
0–5	50 (7.55%)
6–10	61 (9.21%)
10–20	124 (18.73%)
>20	403 (60.88%)
NA	24 (3.63%)
Practice settings	
Academic	40 (6.04%)
Private	354 (53.47%)
Hospital-based	215 (32.48%)
NA	53 (8.01%)

**Table 2:** Analysis based on responses to the statement “Feel adequately updated on myopia prevention” showing that those who are definitely updated on myopia prevention use combined treatments if the first strategy fails. *p*-value calculated via Chi-square test. The reported significance level (*p* < 0.001) remains robust after accounting for multiple comparisons (Bonferroni adjustment).

Questions	Definetly No	More No Than Yes	More Yes Than No	Definetly Yes	Don’t Know/No Answer	*p*-Value
If first strategy fails, then?						<0.001
Propose second treatment	5 (25%)	10 (9.17%)	30 (9.84%)	16 (7.44%)	1 (7.69%)	
Combine with first treatment	9 (45%)	64 (58.72%)	222 (72.79%)	162 (75.35%)	8 (61.54%)	
Refer to secondary care	1 (5%)	9 (8.26%)	19 (6.23%)	3 (1.4%)	0 (0%)	
Do not initiate further treatment	5 (25%)	26 (23.85%)	28 (9.18%)	30 (13.95%)	4 (30.77%)	
Increase atropine concentration	0 (0%)	0 (0%)	6 (1.97%)	4 (1.86%)	0 (0%)	

## Data Availability

The raw data supporting the conclusions of this article will be made available by the authors, without undue reservation.
